# Effects of transitional care interventions on rehospitalization, functional outcomes, and quality of life in stroke survivors: an updated systematic review and meta-analysis of randomized controlled trials

**DOI:** 10.3389/fneur.2026.1769301

**Published:** 2026-06-23

**Authors:** Xiaoqin Yang, Yan Han, Yuntian Liu, Na Zhao, Yan He, Feipeng Li

**Affiliations:** 1 Affiliated Baotou Clinical College, Inner Mongolia Medical University, Hohhot, China; 2 Department of Neurology, Inner Mongolia Baotou City Central Hospital, Baotou, China; 3 Nursing Department, Ordos Center Hospital, Ordos, China; 4 Department of Nursing Department, Baotou Central Hospital, Inner Mongolia, China

**Keywords:** activities of daily living, continuity of care, meta-analysis, quality of life, rehospitalization, stroke, systematic review, transitional care

## Abstract

**Objective:**

To evaluate the effects of transitional care interventions on rehospitalization, functional outcomes, quality of life, mortality, and disability in stroke survivors.

**Methods:**

This systematic review and meta-analysis was conducted in accordance with the Preferred Reporting Items for Systematic Reviews and Meta-Analyses 2020 statement and registered in PROSPERO (CRD420251266063). PubMed, Web of Science, Embase, the Cochrane Library, and CINAHL were searched from inception to May 13, 2026. Additional searches were conducted in ClinicalTrials.gov, Google Scholar, and grey literature sources. Randomized controlled trials comparing transitional care interventions with usual care in adult stroke survivors were included. Dichotomous outcomes were pooled as risk ratios (RRs), and continuous outcomes were pooled as standardized mean differences (SMDs), both with 95% confidence intervals (CIs). Subgroup analyses were conducted according to follow-up duration. Sensitivity analyses, small-study-effect assessment, and GRADE certainty assessment were performed where appropriate.

**Results:**

Twenty-four randomized controlled trials involving 3,520 stroke survivors were included. Transitional care interventions reduced overall rehospitalization compared with usual care (RR = 0.59, 95% CI 0.40–0.88), with a more consistent effect within 3 months after discharge (RR = 0.49, 95% CI 0.29–0.82). Transitional care interventions improved activities of daily living (ADL) in the primary analysis (SMD = 0.43, 95% CI 0.20–0.67), although heterogeneity was substantial; after sensitivity analysis, the effect remained statistically significant but was attenuated (SMD = 0.22, 95% CI 0.07–0.37). Quality of life (QoL) improved overall (SMD = 0.67, 95% CI 0.37–0.96), particularly within 3 months (SMD = 0.74, 95% CI 0.54–0.94). No significant mortality reduction was observed (RR = 0.86, 95% CI 0.53–1.39). Transitional care interventions were associated with lower short-term disability measured by the modified Rankin Scale (mRS; SMD = −0.59, 95% CI −0.99 to −0.19), but this finding was limited by substantial heterogeneity. The certainty of evidence was moderate for short-term rehospitalization and short-term QoL, and low for ADL, mortality, and disability.

**Conclusion:**

Transitional care interventions probably reduce short-term rehospitalization and improve short-term QoL in stroke survivors. They may also improve ADL and short-term disability, but these findings should be interpreted cautiously because of heterogeneity, risk of bias, and possible small-study effects. Current evidence does not establish a clear mortality benefit.

## 1 Introduction

Stroke remains one of the leading causes of death and long-term disability worldwide, with a burden that extends well beyond acute hospitalization (1). Improvements in emergency care, reperfusion therapy, stroke-unit management, and early rehabilitation have increased survival; however, many stroke survivors continue to experience persistent limitations in mobility, self-care, communication, cognition, and social participation after discharge (2, 3). The transition from hospital to home is therefore a clinically vulnerable period. During this phase, stroke survivors and their caregivers are expected to manage medication regimens, rehabilitation exercises, secondary prevention, emotional adjustment, and navigation of community-based services, often with limited professional support. Gaps in post-discharge care may contribute to functional deterioration, reduced QoL, preventable complications, and unplanned healthcare use (4, 5).

Transitional care refers to coordinated, time-limited care processes intended to maintain continuity and safety when patients move between care settings, particularly from hospital to home (6, 7). In stroke care, transitional care interventions may include individualized discharge planning, structured follow-up, home visits, telephone or digital monitoring, rehabilitation guidance, medication and vascular risk-factor management, caregiver education, self-management support, and linkage to outpatient or community resources. These components are designed to bridge the discontinuity between inpatient stroke management and recovery in real-world home and community environments. By extending clinical oversight beyond discharge, transitional care may reduce fragmentation, support early recognition of post-discharge problems, and improve survivors’ engagement in rehabilitation and secondary prevention.

Several systematic reviews have evaluated transitional care, early supported discharge, and post-discharge rehabilitation in stroke survivors, reporting potential benefits for mortality, ADL, functional recovery, rehospitalization-related outcomes, or QoL (8–10). Nevertheless, important uncertainties remain. First, previous reviews have often synthesized clinically diverse interventions without clearly distinguishing short-term transition effects from longer-term outcomes. Second, outcome classification has not always been consistent; in particular, ADL has sometimes been discussed together with patient-reported outcomes despite being a functional or assessor-dependent measure. Third, certainty of evidence has not always been graded across major outcomes, limiting the ability to determine whether statistically significant pooled estimates provide a reliable basis for clinical recommendations. Finally, heterogeneity has frequently been acknowledged but not sufficiently explored, especially for functional and QoL outcomes.

To address these gaps, we conducted an updated systematic review and meta-analysis of randomized controlled trials evaluating transitional care interventions for stroke survivors. This review updates the evidence base, classifies outcomes into clinical, functional, and QoL domains, examines follow-up duration as a potential source of heterogeneity, applies sensitivity and small-study-effect assessments where appropriate, and evaluates certainty of evidence using the GRADE approach. The primary objective was to determine whether transitional care reduces rehospitalization after stroke. Secondary objectives were to evaluate its effects on ADL, QoL, mortality, and disability, and to clarify the certainty and clinical interpretability of the available evidence.

## 2 Methods

### 2.1 Study design and protocol registration

This systematic review and meta-analysis was conducted in accordance with the Preferred Reporting Items for Systematic Reviews and Meta-Analyses 2020 statement (11). The review protocol was registered in PROSPERO (CRD420251266063). The registered protocol was used as the methodological framework for this updated review. Prespecified eligibility criteria and the core review question were retained. During the update process, several protocol amendments were made to strengthen transparency and interpretability: the search was updated; outcome domains were refined into clinical, functional, and QoL categories; subgroup analyses based on follow-up duration were added; sensitivity analyses were conducted for outcomes with substantial heterogeneity; small-study effects were assessed where methodologically appropriate; and certainty of evidence was evaluated using the GRADE approach. These amendments are reported transparently in the Methods and Results sections and were considered when interpreting the findings.

### 2.2 Search strategy

A comprehensive literature search was conducted in PubMed, Web of Science, Embase, the Cochrane Library, and CINAHL from database inception to May 13, 2026. This date represents the final search date for the updated review. Additional searches were conducted in ClinicalTrials.gov, Google Scholar, and grey literature sources to identify potentially eligible published, ongoing, or unpublished studies. The search strategy combined controlled vocabulary terms and free-text terms related to stroke and transitional care. Stroke-related terms included “stroke,” “ischemic stroke,” “hemorrhagic stroke,” “cerebral infarction,” “brain infarction,” and “cerebral hemorrhage.” Transitional care-related terms included “transitional care,” “care transition,” “hospital to home,” “post discharge care,” and “supported discharge.” The search strategies were adapted for each database according to its indexing system and syntax. The detailed search strategies for all databases are provided in Supplementary Table S1.

Only English-language reports were included. This language restriction was applied because of feasibility constraints in full-text assessment and data extraction; the potential for language bias was considered when interpreting the findings.

### 2.3 Eligibility criteria

Studies were selected according to the PICOS framework.

#### 2.3.1 Participants

Eligible participants were adult stroke survivors aged 18 years or older who were discharged from hospital or were transitioning from acute or inpatient care to home, community, outpatient, or rehabilitation settings. Studies involving mixed neurological or older adult populations were eligible only when stroke-specific data could be extracted.

#### 2.3.2 Interventions

Interventions were eligible if they included structured transitional care, continuity-of-care, early supported discharge, post-discharge care management, hospital-to-home follow-up, or home-based transitional rehabilitation for stroke survivors. Eligible interventions had to include a clear care-transition component delivered during the discharge period, after discharge, or across the hospital-to-home transition. Intervention components could include discharge planning, individualized needs assessment, home visits, telephone follow-up, multidisciplinary coordination, health education, medication or risk-factor management, rehabilitation guidance, caregiver training, self-management support, and linkage to community or outpatient services.

#### 2.3.3 Comparators

Comparators included usual discharge care, standard rehabilitation, routine outpatient follow-up, conventional community care, or other standard-care conditions as defined by the original studies.

#### 2.3.4 Outcomes

Eligible outcomes included clinical outcomes, functional outcomes, QoL outcomes, and other patient-centered outcomes. Clinical outcomes included rehospitalization, mortality, recurrent admission, falls, institutionalization, skilled nursing facility admission, and other adverse events. Functional outcomes included ADL, disability, motor function, neurological status, and functional independence. QoL outcomes included generic or stroke-specific QoL measures. Other patient-centered outcomes, when available, included self-efficacy, psychological outcomes, satisfaction, caregiver-related outcomes, and health-service utilization. Studies were eligible if they reported at least one outcome of interest; they were not required to report both primary and secondary outcomes.

#### 2.3.5 Study design

Only randomized controlled trials were included. Non-randomized studies, observational studies, single-arm studies, case reports, reviews, letters, editorials, expert consensus articles, conference abstracts without sufficient outcome data, duplicate publications, and studies without extractable outcome data were excluded. When multiple reports were based on the same trial population, the report with the most complete and relevant outcome data was retained.

### 2.4 Study selection

All records retrieved from the searches were imported into reference-management software, and duplicates were removed. Two reviewers independently screened titles and abstracts for potential eligibility. Full texts of potentially relevant reports were then obtained and assessed independently by the same reviewers according to the predefined eligibility criteria. Disagreements during title/abstract screening or full-text assessment were resolved through discussion. If consensus could not be reached, a third reviewer was consulted. Reasons for exclusion at the full-text stage were recorded and summarized in the PRISMA flow diagram.

### 2.5 Data extraction

A standardized data extraction form was developed before formal data extraction and pilot-tested on a sample of included studies. After pilot testing, the extraction form was refined by adding fields for intervention provider, delivery mode, intervention duration, contact frequency, caregiver involvement, rehabilitation content, outcome domain, and follow-up category. The form was refined to ensure consistent extraction of study characteristics, intervention features, comparator details, outcome definitions, follow-up time points, and numerical data required for meta-analysis. The final extraction form included the following fields: first author, publication year, country or region, study design, sample size, participant characteristics, stroke type where reported, intervention components, intervention provider, delivery mode, intervention duration, contact frequency where available, caregiver involvement, rehabilitation content, comparator characteristics, follow-up time points, outcome domain, measurement instrument, and numerical outcome data. To improve transparency in the description of complex interventions, an intervention-component matrix was prepared to summarize provider type, delivery mode, home visits, telephone or digital follow-up, active rehabilitation content, caregiver involvement, intervention duration, and follow-up timing.

Two reviewers independently extracted data from each included study, and discrepancies were resolved by discussion or consultation with a third reviewer. For dichotomous outcomes, numbers of events and total participants in each group were extracted. For continuous outcomes, means, standard deviations, and sample sizes were extracted. When studies reported standard errors, confidence intervals, medians with interquartile ranges, or other summary statistics, these were converted to means and standard deviations where appropriate using established methods. If necessary data were unavailable or could not be reliably converted, the outcome was excluded from quantitative synthesis and described narratively where relevant.

When multiple follow-up time points were reported, outcomes were extracted according to predefined follow-up categories: short-term follow-up, defined as 3 months or less, and longer-term follow-up, defined as more than 3 months. This classification was used to explore whether intervention effects differed according to follow-up duration.

### 2.6 Risk of bias assessment

The methodological quality of the included randomized controlled trials was assessed using the Cochrane Risk of Bias tool (12). The following domains were evaluated: random sequence generation, allocation concealment, blinding of participants and personnel, blinding of outcome assessment, incomplete outcome data, selective reporting, and other potential sources of bias. Each domain was judged as low, unclear, or high risk of bias.

Two reviewers independently assessed risk of bias, and disagreements were resolved through discussion or adjudication by a third reviewer. No study was excluded solely on the basis of risk of bias. Instead, risk-of-bias judgments were considered in the interpretation of pooled findings and incorporated into the GRADE certainty-of-evidence assessment. Because transitional care interventions are complex behavioral and service-delivery interventions, blinding of participants and personnel was not always feasible; therefore, its potential impact was interpreted differently for objective outcomes, such as rehospitalization and mortality, and subjective or assessor-dependent outcomes, such as QoL, ADL, and disability.

### 2.7 Statistical analysis

Meta-analyses were performed using Review Manager version 5.4.1 and R version 4.6.0. Dichotomous outcomes were summarized using RRs with 95% CIs. Continuous outcomes were summarized using SMDs with 95% CIs because different instruments and scoring systems were used across studies.

Random-effects models were used for the primary analyses because clinical and methodological heterogeneity was expected across studies, including differences in transitional care components, intervention intensity, provider type, healthcare setting, comparator content, outcome instruments, and follow-up duration. Statistical heterogeneity was assessed using Cochran’s *Q* test and the *I*^2^ statistic. *I*^2^ values greater than 50% were considered to indicate substantial heterogeneity.

To explore potential sources of heterogeneity, subgroup analyses were conducted according to follow-up duration, categorized as short-term follow-up of 3 months or less and longer-term follow-up of more than 3 months. Because the number of studies within each intervention-component category was small and several components overlapped within multicomponent interventions, subgroup analyses by provider type, delivery mode, or individual intervention component were not considered statistically reliable. Sensitivity analyses were performed for outcomes with substantial heterogeneity by excluding influential studies with extreme effect estimates or disproportionate contributions to heterogeneity.

For outcomes with at least 10 studies, funnel plots and Egger’s test were used to assess potential small-study effects or publication bias. Publication bias tests were not performed for outcomes with fewer than 10 studies because such tests have limited power and may yield unreliable conclusions when the number of studies is small. A two-sided *p*-value less than 0.05 was considered statistically significant.

### 2.8 Certainty of evidence

The certainty of evidence for the main outcomes was assessed using the Grading of Recommendations Assessment, Development and Evaluation approach (13). Evidence certainty was rated as high, moderate, low, or very low according to the following domains: risk of bias, inconsistency, indirectness, imprecision, and publication bias. Summary of Findings tables were prepared for clinically important outcomes, including short-term rehospitalization, ADL, short-term QoL, mortality, and disability measured by the mRS. The certainty ratings were used to guide the interpretation of findings and to avoid overstatement of conclusions, particularly for outcomes with high heterogeneity, small numbers of studies, wide CIs, or possible small-study effects.

## 3 Results

### 3.1 Study selection

**Figure 1:**
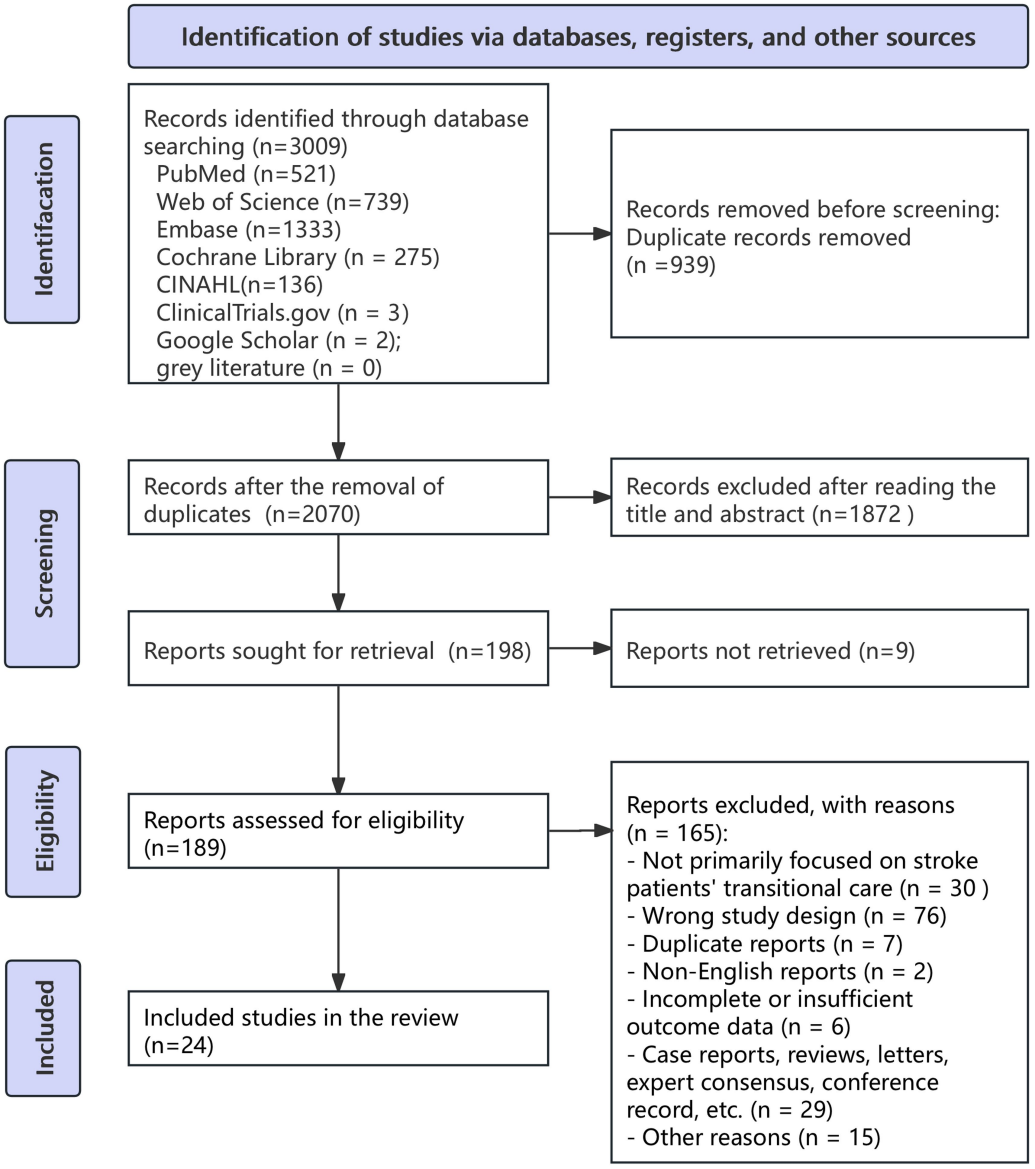
PRISMA flow diagram.

The study selection process is shown in the PRISMA flow diagram (Figure 1). A total of 3,009 records were identified, and 24 randomized controlled trials were ultimately included after duplicate removal, title and abstract screening, and full-text eligibility assessment. Although the final search was conducted on May 13, 2026, no additional eligible randomized controlled trial published between 2025 and the final search date met the inclusion criteria.

### 3.2 Characteristics of included studies

The main characteristics of the 24 included randomized controlled trials are summarized in Table 1. The studies were published between 1997 and 2024 and included a total of 3,520 stroke survivors, with 1,763 participants allocated to transitional care interventions and 1,757 allocated to control conditions. Individual sample sizes ranged from 60 to 380 participants. All included studies used randomized controlled designs and evaluated structured post-discharge or hospital-to-home transitional care models for stroke survivors.

**Table 1:** Characteristics of the included randomized controlled trials.

Study	Design	Participants	Total (*n*)	IG (*n*)	CG (*n*)	Intervention	Control	Follow-up duration	Outcomes
Krauss et al. (17) USA	RCT	Stroke	116	58	58	A 12-week structured home-based transitional care program, including a pre-discharge session, individualized assessment, six post-discharge home visits, six telephone follow-up calls, hotline support, health education and counselling, medication and risk-factor management, structured home-based rehabilitation training, self-management toolkit, caregiver involvement, and continuous monitoring of rehabilitation progress.	Usual discharge care, outpatient follow-up, access to medical/emergency services, and one nurse-initiated telephone call	6 months	Modified Barthel Index (MBI); EQ-5D-5L; EQ-VAS; Stroke Impact Scale (SIS); Self-Efficacy for Managing Chronic Disease 6-item Scale (SEMCD-6)
García-Pérez et al. (18) ESP	RCT	Stroke	183	85	98	A home-based transition program including a pre-discharge home assessment, home modifications to reduce environmental barriers, adaptive equipment, self-management training, problem-solving strategies for daily activities, four post-discharge home visits, and booster sessions at 4–5 months.	Attention-control stroke education with the same number of visits, covering stroke symptoms, risk factors, stroke recurrence prevention, nutrition, and booster education sessions.	30 days/study completion	30-day rehospitalization; skilled nursing facility (SNF) admission; death; falls; fall rate; Geriatric Depression Scale–Short Form (GDS-SF)
Li et al. (19) CHN	RCT	Stroke	60	30	30	An early occupational therapy discharge-transition program including initial evaluation, individualized goal setting, one hospital-based session, post-discharge home visit, weekly telephone follow-up, one home session 1 month after discharge, home-based rehabilitation guidance, patient/caregiver education, and final evaluation at 3 months.	Conventional rehabilitation and care within the public healthcare system, including inpatient physiotherapy when referred and outpatient physiotherapy or occupational therapy depending on rehabilitation physician assessment.	3 months	Barthel Index (BI); Stroke and Aphasia Quality of Life Scale-39 (SAQOL-39); modified Rankin Scale (mRS); Montreal Cognitive Assessment (MoCA); Berg Balance Scale (BBS); Timed Up and Go (TUG); NIHSS; Beck Depression Inventory-II (BDI-II); Hamilton Anxiety Scale (HAM-A)
Björkdahl et al. (20) SWE	RCT	Stroke	120	60	60	An extended care program including in-hospital health education, discharge preparation, rehabilitation diary, individualized discharge planning, post-discharge health goals, WeChat and telephone consultation, telephone follow-up, home visits, home environment assessment, ADL training, home rehabilitation guidance, medication adherence, risk-factor management, diet and lifestyle guidance, psychological support, caregiver guidance, scheduled hospital review, and adjustment of home rehabilitation plans.	Routine specialized care and systematic rehabilitation during hospitalization; verbal discharge guidance on medication, blood pressure monitoring, rehabilitation training, simple psychological counseling, diet, lifestyle, and recurrence prevention; no routine home-based extended care after discharge.	3 months	Barthel Index (BI); Fugl-Meyer Motor Function Assessment (FMA)
Kam Yuet Wong et al. (21) CHN	RCT	Stroke	104	52	52	A 4-week very early supported discharge program comprising home-based, goal-oriented rehabilitation 2–4 times per week, individualized ADL training, personal care, transfers, household and leisure activities, and support for activity-based goals set before discharge.	Usual discharge routine, including referral to outpatient primary care, community rehabilitation, nursing-home rehabilitation, or home-based rehabilitation when needed.	12 months	ADL Taxonomy (B-ADL and I-ADL); Barthel Index (BI); Stroke Impact Scale (SIS-ADL and SIS-Mobility); modified Rankin Scale (mRS); Montreal Cognitive Assessment (MoCA).
Lin et al. (22) CHN	RCT	Stroke	140	70	70	A 12-week nurse-led health coaching transitional care programme including transitional goal setting, self-care skill enhancement, home-environment modification, physical-function enhancement, medication management, prevention of stroke-related adverse events, weekly telephone support, and bi-weekly face-to-face outpatient coaching.	Usual transitional discharge plan, including verbal health education before discharge and two post-discharge telephone follow-up calls.	6 months	Stroke Self-Efficacy Questionnaire (SSEQ); Stroke-Specific Quality of Life Scale-12 (SSQoL-12); Modified Caregiver Strain Index (CSI); unplanned hospital readmissions.
Mohammadi et al. (23) IRN	RCT	Stroke	67	31	36	A partnership care model including partnership education sessions, assessment of care needs, motivational and readiness-building sessions, patient/family participation, education on stroke status, disease nature, causes and complications, diet and activity planning, medication education, monthly follow-up partnership visits for 6 months, blood pressure monitoring, problem review, evaluation of previous care, new care/nutrition prescriptions, and referral to rehabilitation services when needed.	Usual rehabilitation-center care and routine medical visits; patients received routine hospital care, physician visits, drug/nutrition prescriptions, and referral to specialized rehabilitation services when needed, but no structured partnership education or continuous follow-up model.	6 months	Stroke-Specific Quality of Life Scale (SS-QOL); Lawton Activities of Daily Living / Instrumental Activities of Daily Living Scale (Lawton ADL/IADL)
Deng et al. (24) CHN	RCT	Stroke	98	49	49	An 8-week integrated transitional care program including home-based rehabilitation, medication reconciliation, stroke risk-factor control, self-management education, stroke warning-sign education, periodic telephone follow-up, specialist consultation, and encouragement of continued home rehabilitation training.	Usual post-discharge care based on secondary stroke prevention, including detection and control of risk factors and medication therapy; no multidisciplinary rehabilitation treatment until outcome assessments.	8 weeks	Modified Barthel Index (MBI); SF-36 Physical Component Summary (PCS); SF-36 Mental Component Summary (MCS); Caregiver Strain Index (CSI)
Chu et al. (25) CHN	RCT	Stroke	61	31	30	A nurse-trained, family member-delivered rehabilitation programme including in-hospital caregiver training, individualized rehabilitation planning, mobility training, self-care training, bowel/toilet control training, rehabilitation manual, mobile app-assisted assessment and guidance, and post-discharge telephone follow-up at weeks 2, 4, and 8.	Routine care, including rehabilitation during hospitalization, discharge guidance, and routine post-discharge follow-up.	6 months	Barthel Index (BI); modified Rankin Scale (mRS); EQ-5D; Caregiver Burden Inventory (CBI); functional walking grade; hospitalization;
Bragstad et al. (26) NOR	RCT	Stroke	322	166	156	A dialogue-based psychosocial intervention comprising eight individualized 1–1.5 h sessions delivered in the community, mainly at home, during the first 6 months after stroke; sessions used stroke-related topics, worksheets, guided dialogue, coping support, and individualized adaptation of topics and timing.	Standard stroke treatment and usual rehabilitation services according to Norwegian stroke guidelines, including access to physical therapy, occupational therapy, speech therapy, and/or home nursing care according to need and availability.	12 months	General Health Questionnaire-28 (GHQ-28); Stroke and Aphasia Quality of Life Scale-39 g (SAQOL-39 g); Sense of Coherence Scale-13 (SOC-13); Yale single-item depression question; fatigue; NIHSS; Mini-Mental State Examination (MMSE)
Rafsten et al. (27) SWE	RCT	Stroke	140	69	71	A very early supported discharge program including pre-discharge goal setting, individualized home-based rehabilitation, two to four weekly visits by physiotherapy/occupational therapy, one to two visits by stroke nurse, activity-based training, adaptation strategies, support for patients and next of kin, and referral to outpatient rehabilitation when needed; maximum duration 4 weeks after discharge.	Usual discharge routine, including referral to outpatient rehabilitation, home care service, physiotherapy, occupational therapy, speech therapy, or other support when needed; no structured goal-setting meeting or stroke-unit multidisciplinary home follow-up.	12 months	Barthel Index (BI); modified Rankin Scale (mRS); Hospital Anxiety and Depression Scale-Anxiety subscale (HADS-A); Hospital Anxiety and Depression Scale-Depression subscale (HADS-D); Montreal Cognitive Assessment (MoCA); NIHSS; Fugl-Meyer Assessment (FMA); length of hospital stay; continued rehabilitation use
Qian et al. (28) CHN	RCT	Stroke	72	35	37	A 5-week transitional nursing care programme including pre-discharge health education, Omaha-based nursing evaluation, compliance diary, rehabilitation training guidance, diet and lifestyle guidance, medication and follow-up education, telephone follow-up at 1 week after discharge, and home visit at 4 weeks after discharge with emphasis on home rehabilitation exercises.	Conventional nursing care.	12 weeks	Barthel Index (BI); Nine-Hole Peg Test (NHPT); subjective quality of life (SQOL); patient compliance; re-hospitalization; emergency visits; outpatient visits
Chen et al. (29) CHN	RCT	Stroke	144	72	72	A patient-centered self-management empowerment intervention including individualized assessment, bedside self-management education, short- and long-term goal setting, problem-solving training, complication prevention, functional rehabilitation guidance, self-health monitoring, small-group DVD-based education, peer experience sharing, discharge planning, collaborative action planning, and four weekly post-discharge telephone follow-ups.	Routine care, including conventional nursing, unstructured health education, post-discharge medical follow-up, and telephone calls for general social chatting to balance professional contact.	3 months	Stroke Self-Efficacy Questionnaire (SSEQ); Barthel Index (BI); rehospitalization
Santana et al. (30) POR	RCT	Stroke	190	95	95	An early home-supported discharge service starting in the stroke unit and continuing at home, including case management, individualized home rehabilitation planning, approximately eight home-based training sessions for up to one month, home adaptations/aids when feasible, education for patients and carers, and rehabilitation focused on meaningful daily activities, personal care, outdoor walking, shopping and leisure activities.	Usual stroke-unit care and standard rehabilitation available in the region, including discharge home with or without ambulatory rehabilitation, or transfer to convalescence/inpatient rehabilitation units when prescribed.	12 months	Functional Independence Measure (FIM); Barthel Index (BI); Frenchay Activities Index (FAI); WHOQOL-BREF; Short Form-6D (SF-6D); length of stay; discharge to rehabilitation unit; death
Wong and Yeung (31) CHN	RCT	Stroke	108	54	54	A 4-week transitional care programme based on the Omaha System, including pre-discharge holistic assessment and care planning, entrance and exit family meetings, weekly home visits, weekly telephone follow-up, health teaching and counselling, symptom management, stroke recurrence prevention, physical-function and self-care guidance, medication/diet adherence support, resilience-building, emotion management, case management and referral when needed.	Routine hospital-based physical training programme within the first 3 weeks after discharge; therapist assessment at first training session with follow-up sessions if appropriate.	8 weeks	Modified Barthel Index (MBI); SF-36 Physical Component Summary/Mental Component Summary (SF-36 PCS/MCS); WHOQOL-SRPB-HK; Center for Epidemiological Studies Depression Scale (CES-D); hospital readmission; emergency room visits
Rasmussen et al. (32) DEN	RCT	Stroke	71	38	33	A home-based rehabilitation delivered before and after discharge, including transporting inpatients home for training 1–3 times/week, ADL and physical training in the patient’s own home, assessment of home environment and aids, family involvement, discharge coordination with municipality professionals, written home-training plans, post-discharge rehabilitation 1–5 days/week for up to 4 weeks, lifestyle/medication/sequelae counselling, and transition planning to municipal rehabilitation when needed.	Standard stroke-unit treatment and rehabilitation according to usual guidelines, followed by standard municipal rehabilitation and home-care services according to individual needs.	150 days	modified Barthel-100 ADL Index; modified Rankin Scale (mRS); Motor Assessment Scale (MAS); CT-50 Cognitive Test; EuroQol-5D; length of hospital stay
Hofstad et al. (33) NOR	RCT	Stroke	202	103	99	Early supported discharge with a multidisciplinary ambulatory team, early discharge planning, coordinated hospital-community transition, and community day-unit rehabilitation for up to 5 weeks, with 3- and 6-month follow-up controls.	Treatment as usual, including institutional stay if needed and/or municipal physiotherapy as needed, usually 0–2 h/week; no ESD team intervention except outcome testing.	6 months	Barthel Index (BI); modified Rankin Scale (mRS); NIHSS; discharge destination; days in stroke unit; days in rehabilitation/institution; total institutional days
Chaiyawat and Kulkantrakorn (34) THA	RCT	Stroke	60	30	30	A 6-month individualized home rehabilitation programme including monthly home visits by a physical therapist, individualized mobility and ADL training, caregiver counselling, audiovisual rehabilitation materials, passive/active/resistance exercises, ADL practice, cane/wheelchair use, caregiver-assisted practice and training diary.	Usual care, including inpatient physical therapy, pre-discharge instructions for home rehabilitation, and outpatient rehabilitation or home exercise advice at physicians’ discretion; no scheduled home visits.	24 months	Barthel Index (BI); modified Rankin Scale (mRS); EQ-5D utility index; good functional outcome; adverse events
Chalermwannapong et al. (35) THA	RCT	Stroke	67	33	34	A transitional care programme including in-hospital discharge planning, individualized assessment, education, ADL and mobility training, caregiver training, complication prevention, stress management, home safety/home modification guidance, booklet/video materials, two post-discharge home visits, and two telephone visits during the first 4 weeks after discharge.	Usual hospital care, including routine nursing care, general discharge information, physician rounds, and referral to physical therapy, occupational therapy, dietitian or community home visit if ordered.	12 weeks	Modified Barthel Index (MBI); Ferrans and Powers’ Quality of Life Index–Stroke Version (QLI-Stroke)
Allen et al. (36) USA	RCT	Stroke	380	190	190	A 6-month comprehensive postdischarge care-management model including in-home assessment within 1 week after discharge, individualized interdisciplinary care planning, primary-care physician communication, medication reconciliation, risk-factor management, education, social-service linkage, monitoring of common poststroke complications, personalized health record, telephone follow-up and additional home visits as needed.	Organized acute stroke department care with enhanced discharge planning; primary-care physician received written inpatient summary, risk-factor profile, discharge plan, medication list and baseline assessment data; usual postdischarge care from primary-care physician; educational mailings every 2 months.	6 months	NIHSS; Timed Up and Go test (TUG); Physical Performance Test; Stroke-Specific Quality of Life Scale (SS-QOL); Center for Epidemiological Studies Depression Scale (CES-D); institution time or death; falls; medication compliance;
Askim et al. (37) NOR	RCT	Stroke	62	31	31	Extended stroke unit service with early supported discharge, coordinated by a mobile stroke team, including discharge planning, home-environment assessment, coordination with primary healthcare, telephone follow-up, at least one post-discharge home visit, outpatient or home consultation at 4 weeks, and local patient/family meeting when feasible.	Ordinary stroke unit service, including acute stroke unit treatment and further rehabilitation or follow-up organized by rehabilitation clinics and/or primary healthcare system.	12 months	Barthel Index (BI); modified Rankin Scale (mRS); Nottingham Health Profile (NHP); Caregiver Strain Index (CSI); mortality; length of stay in stroke unit; total institutional stay
Indredavik et al. (38) NOR	RCT	Stroke	320	160	160	Extended stroke unit service including acute stroke-unit treatment, mobile stroke team coordination, early supported discharge, home visit and discharge planning, close cooperation with primary healthcare, rehabilitation at home or day clinic, outpatient consultation 1 month after discharge, secondary prevention review, and patient/family information meeting at 3 months.	Ordinary stroke unit service, including acute and rehabilitation stroke-unit treatment, followed by further inpatient rehabilitation when needed and follow-up organized by rehabilitation clinics and/or primary healthcare.	26 weeks	Barthel Index (BI); Rankin Scale/modified Rankin Scale (RS/mRS); ADL independence (BI ≥95); global independence (RS ≤ 2); discharge destination; living at home; institutionalization; mortality; length of stay in institutions
Andersen et al. (39) DEN	RCT	Stroke	102	54	48	Physician home-visit follow-up after discharge, consisting of three 1-h home visits at 2, 6 and 12 weeks after discharge, focusing on early detection and treatment of complications, maintenance of function, psychological and social adjustment, medication, referral, service liaison and counselling.	Standard aftercare, including outpatient rehabilitation if prescribed, home care to compensate for disability, and usual access to general practitioner and social services.	6 months	Readmission; repeated readmission; stroke-related readmission; death; institutionalization; Barthel Index (BI); Frenchay Activities Index (FAI); Extended Activities of Daily Living (Extended ADL)
Rudd et al. (40) GBR	RCT	Stroke	331	167	164	Early discharge with specialist community rehabilitation for up to 3 months after randomization, including domiciliary physiotherapy, occupational therapy, speech therapy, therapy aide support, individualized care plans, weekly team meetings, equipment provision and consultant-led coordination.	Conventional hospital and community care, including usual inpatient rehabilitation, discharge planning, stroke clinic/day hospital/outpatient physiotherapy, domiciliary therapy and usual community services when indicated.	12 months	Barthel Index (BI); Rivermead Activities of Daily Living Scale; Frenchay Aphasia Screening Test; Hospital Anxiety and Depression Scale (HADS); Caregiver Strain Index (CSI); mortality.

The included studies were conducted across multiple healthcare systems, including China, Norway, the United States, Sweden, Denmark, Thailand, Spain, Iran, Portugal, and the United Kingdom. Although the geographical distribution was broader than in the previous version of the review, the evidence base remained unevenly distributed across regions and healthcare systems.

The interventions varied in provider type, delivery format, intensity, duration, and follow-up schedule. Most interventions were initiated during hospitalization or around discharge and continued after transition to home or community settings. Common components included pre-discharge assessment, individualized discharge planning, home visits, telephone follow-up, structured health education, medication and risk-factor management, functional rehabilitation guidance, self-management support, caregiver training, and coordination with community or outpatient services. Intervention providers included nurses, multidisciplinary stroke teams, physicians, occupational therapists, rehabilitation professionals, and trained family caregivers. To reduce ambiguity in the definition of transitional care, intervention characteristics were summarized in an intervention-component matrix, including provider type, delivery mode, home-visit component, telephone or digital follow-up, rehabilitation content, caregiver involvement, intervention duration, and follow-up timing (Supplementary Table S2).

Control groups generally received usual discharge care, standard rehabilitation, routine outpatient follow-up, or conventional community-based services. The content of usual care varied across studies and healthcare systems, which should be considered when interpreting between-study heterogeneity.

The included trials reported a range of clinical, functional, and patient-centered outcomes. Clinical outcomes included rehospitalization, mortality, recurrent admission, skilled nursing facility admission, institutionalization, falls, and other adverse events. Functional outcomes included ADL, disability, motor function, and neurological status, measured using instruments such as the Barthel Index, Modified Barthel Index, Functional Independence Measure, mRS, Fugl-Meyer Assessment, Motor Assessment Scale, NIH Stroke Scale, and Rivermead Activities of Daily Living Scale. QoL outcomes were assessed using generic and stroke-specific instruments, including EQ-5D, EQ-VAS, SF-36, Stroke-Specific Quality of Life Scale, Stroke Impact Scale, Stroke and Aphasia Quality of Life Scale-39, Nottingham Health Profile, and related scales.

### 3.3 Risk of bias assessment

**Figure 2:**
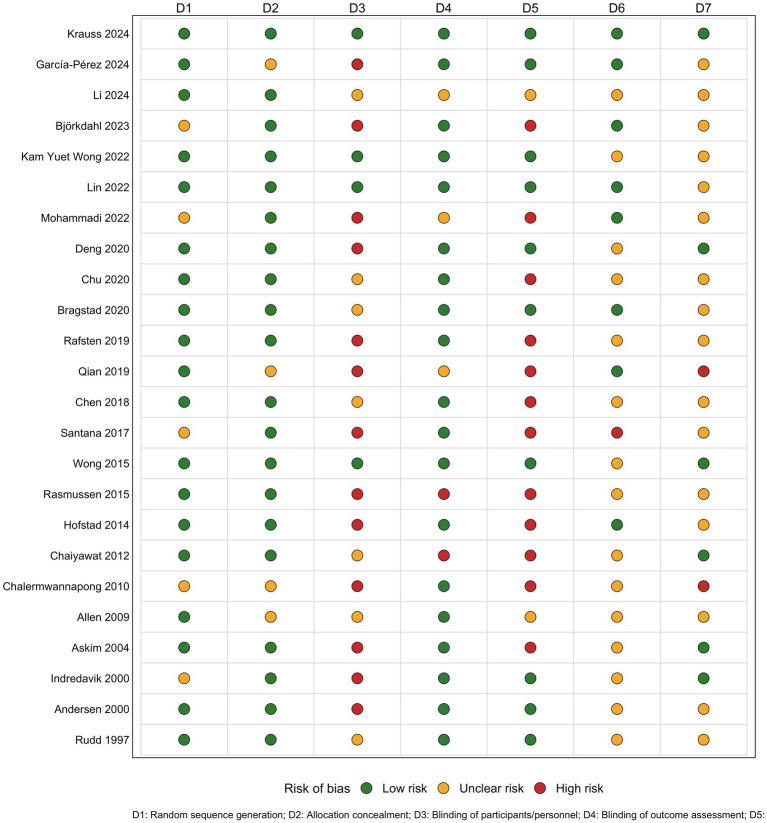
Risk-of-bias traffic light plot. D1, random sequence generation; D2, allocation concealment; D3, blinding of participants/personnel; D4, blinding of outcome assessment; D5, incomplete outcome data; D6, selective reporting; D7, other bias.

**Figure 3:**
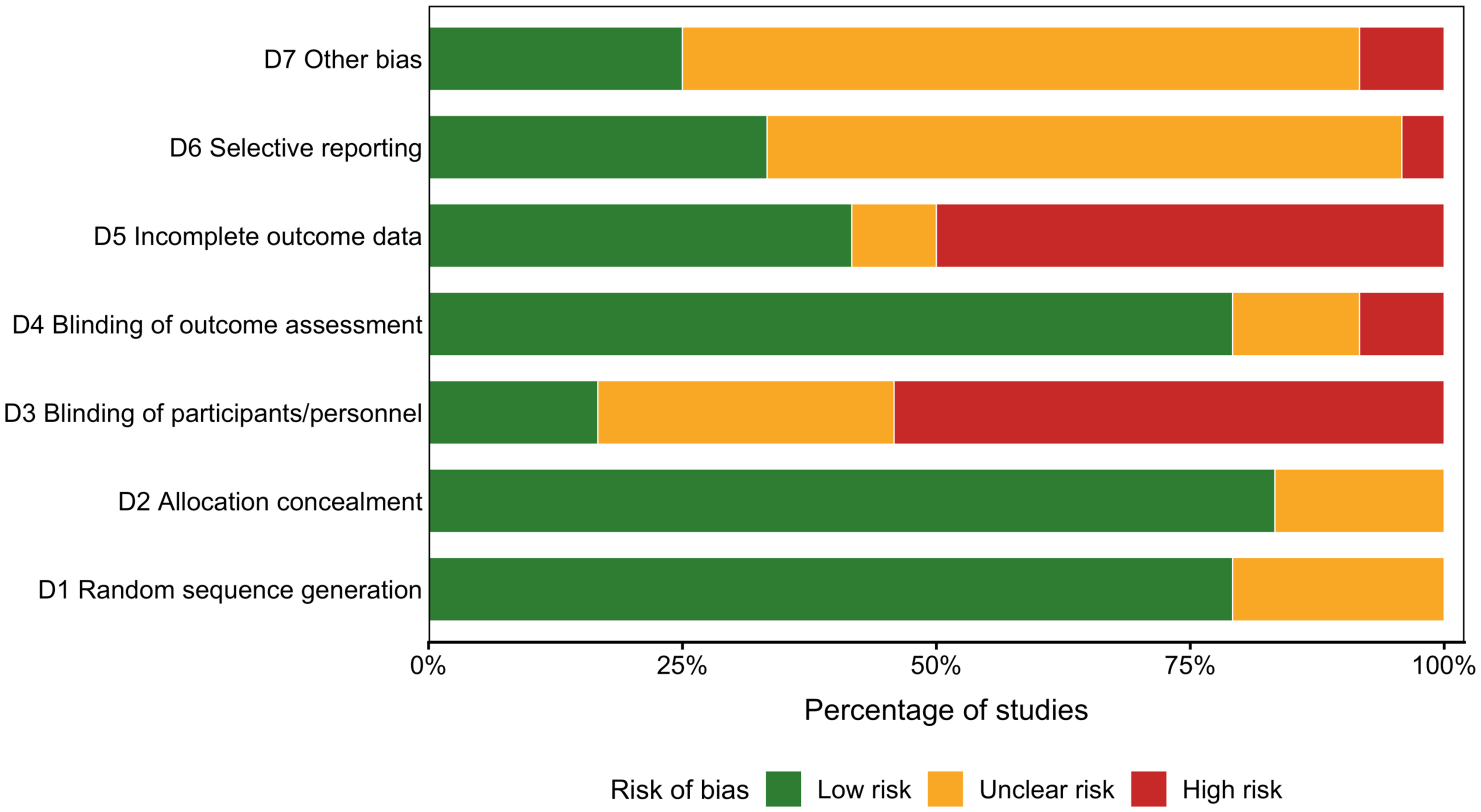
Risk-of-bias summary plot.

The risk of bias assessment is presented in Figures 2, 3. Overall, the included studies showed variable methodological quality. Random sequence generation was judged as low risk in 19 of 24 studies, while 5 studies were rated as unclear risk because the methods used to generate the allocation sequence were insufficiently described. Allocation concealment was judged as low risk in 20 studies and unclear risk in 4 studies.

Blinding of participants and personnel was the most frequent source of bias. Only 4 studies were judged as low risk in this domain, whereas 7 were rated as unclear risk and 13 as high risk. This finding was expected given the nature of transitional care interventions, which usually involve visible components such as home visits, education, rehabilitation guidance, and structured follow-up. Nevertheless, lack of blinding may have influenced subjective or assessor-dependent outcomes, particularly ADL, QoL, and disability measures.

Blinding of outcome assessment was rated as low risk in 19 studies, unclear risk in 3 studies, and high risk in 2 studies. Incomplete outcome data represented another major methodological concern: 10 studies were judged as low risk, 2 as unclear risk, and 12 as high risk. Selective reporting was rated as low risk in 8 studies, unclear risk in 15 studies, and high risk in 1 study, mainly because study protocols or prespecified analysis plans were not always available. Other sources of bias were judged as low risk in 6 studies, unclear risk in 16 studies, and high risk in 2 studies.

No study was excluded solely on the basis of risk of bias. Instead, risk of bias was considered in the interpretation of pooled estimates and incorporated into the certainty-of-evidence assessment.

### 3.4 Effects of transitional care interventions

#### 3.4.1 Rehospitalization

Seven randomized controlled trials involving 1,080 participants reported rehospitalization outcomes. Overall, transitional care interventions were associated with a lower risk of rehospitalization compared with usual care (RR = 0.59, 95% CI 0.40–0.88, *p* = 0.010). Moderate heterogeneity was observed across studies (*I*^2^ = 45%).

**Figure 4:**
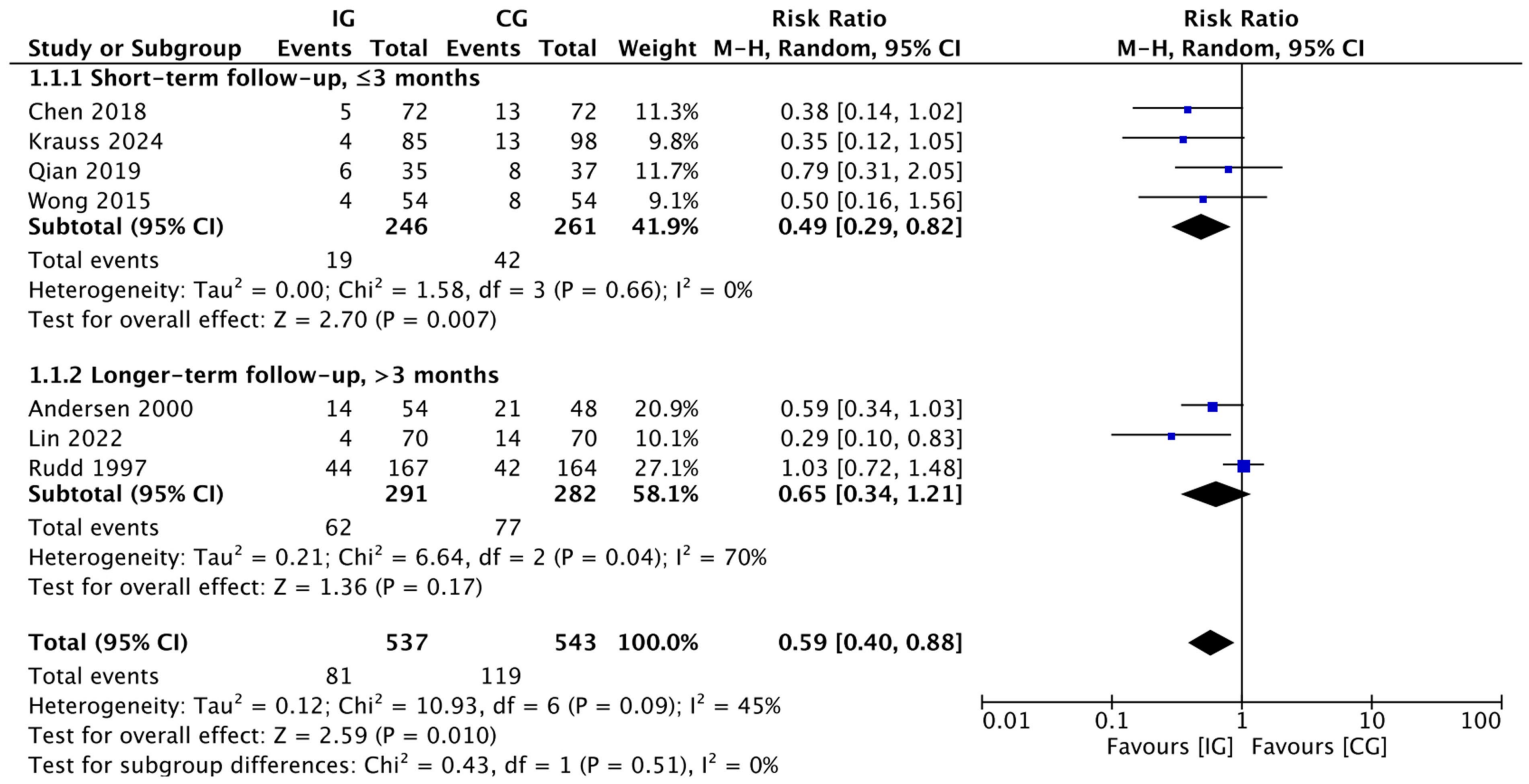
Forest plot of rehospitalization.

Subgroup analysis according to follow-up duration showed that the effect was more evident in studies with short-term follow-up of 3 months or less. Four trials involving 507 participants reported short-term rehospitalization, and the pooled estimate showed a significant reduction in rehospitalization among participants receiving transitional care (RR = 0.49, 95% CI 0.29–0.82, *p* = 0.007), with no observed heterogeneity (*I*^2^ = 0%). In contrast, three trials involving 573 participants reported longer-term rehospitalization beyond 3 months, and the pooled effect was not statistically significant (RR = 0.65, 95% CI 0.34–1.21, *p* = 0.17), with substantial heterogeneity (*I*^2^ = 70%). The test for subgroup differences was not significant, suggesting that the apparent difference between short- and longer-term follow-up should be interpreted cautiously (Figure 4).

#### 3.4.2 Activities of daily living

Sixteen randomized controlled trials involving 1,652 participants reported ADL. In the primary meta-analysis, transitional care interventions were associated with improved ADL performance compared with usual care (SMD = 0.43, 95% CI 0.20–0.67, *p* = 0.0003). However, substantial heterogeneity was present (*I*^2^ = 81%), indicating important variability across studies.

**Figure 5:**
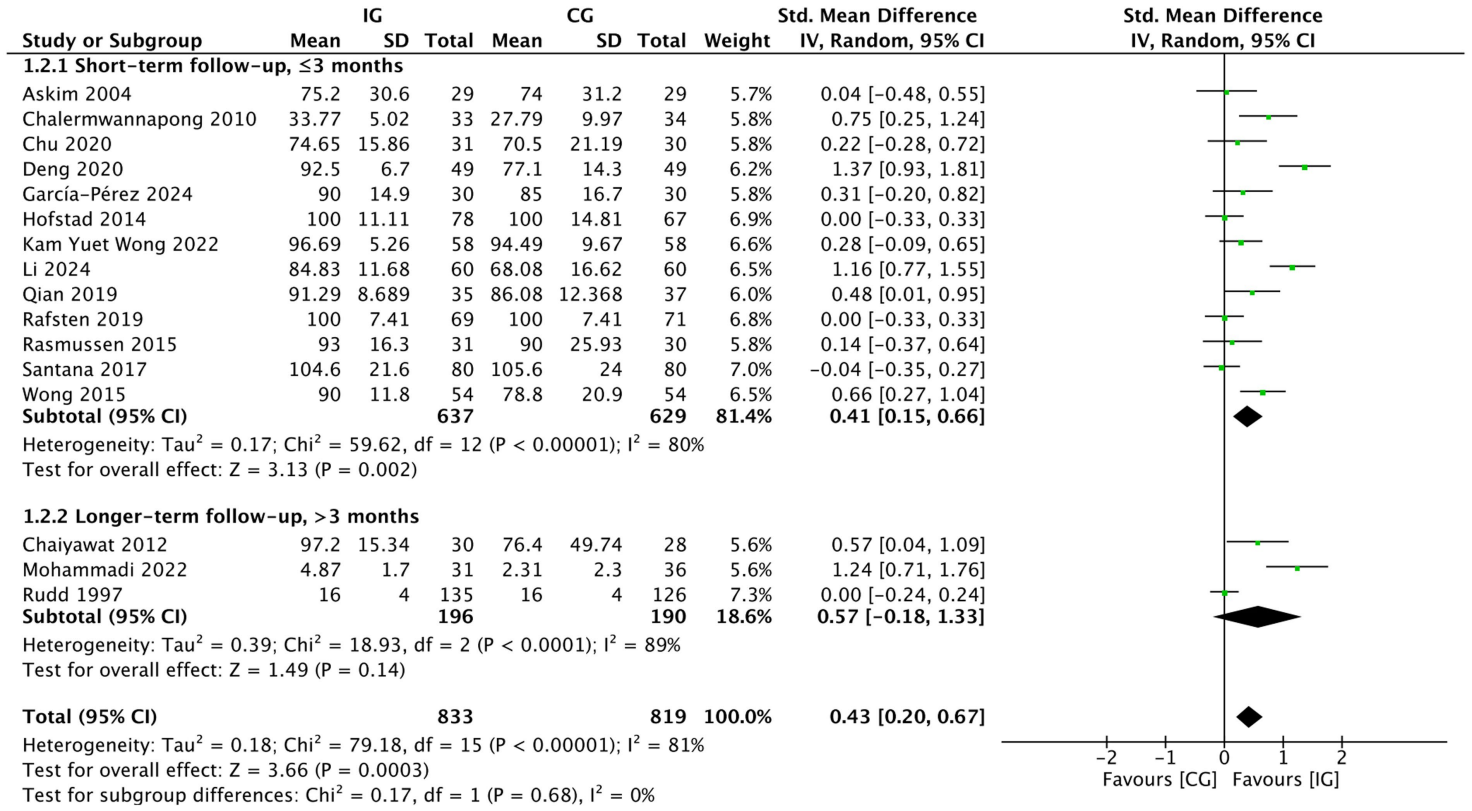
Forest plot of activities of daily living.

In the short-term follow-up subgroup, 13 trials involving 1,266 participants showed a significant improvement in ADL among participants receiving transitional care (SMD = 0.41, 95% CI 0.15–0.66, *p* = 0.002), although heterogeneity remained substantial (*I*^2^ = 80%). In the longer-term subgroup, three trials involving 386 participants showed no statistically significant difference between groups (SMD = 0.57, 95% CI −0.18 to 1.33, *p* = 0.14), with substantial heterogeneity (*I*^2^ = 89%). The test for subgroup differences was not significant (Figure 5).

Given the high heterogeneity in the primary ADL analysis, a sensitivity analysis was conducted by removing influential studies with large effect estimates. After sensitivity analysis, the pooled effect remained statistically significant but was attenuated (13 trials, 1,367 participants; SMD = 0.22, 95% CI 0.07–0.37, *p* = 0.003), and heterogeneity decreased from substantial to moderate (*I*^2^ = 45%). These findings suggest that the direction of effect was robust, but the magnitude of ADL improvement should be interpreted cautiously (Supplementary Figure S1).

#### 3.4.3 Quality of life

Nine randomized controlled trials involving 971 participants reported QoL outcomes. Overall, transitional care interventions were associated with improved QoL compared with usual care (SMD = 0.67, 95% CI 0.37–0.96, *p* < 0.00001), although substantial heterogeneity was observed (*I*^2^ = 78%).

**Figure 6:**
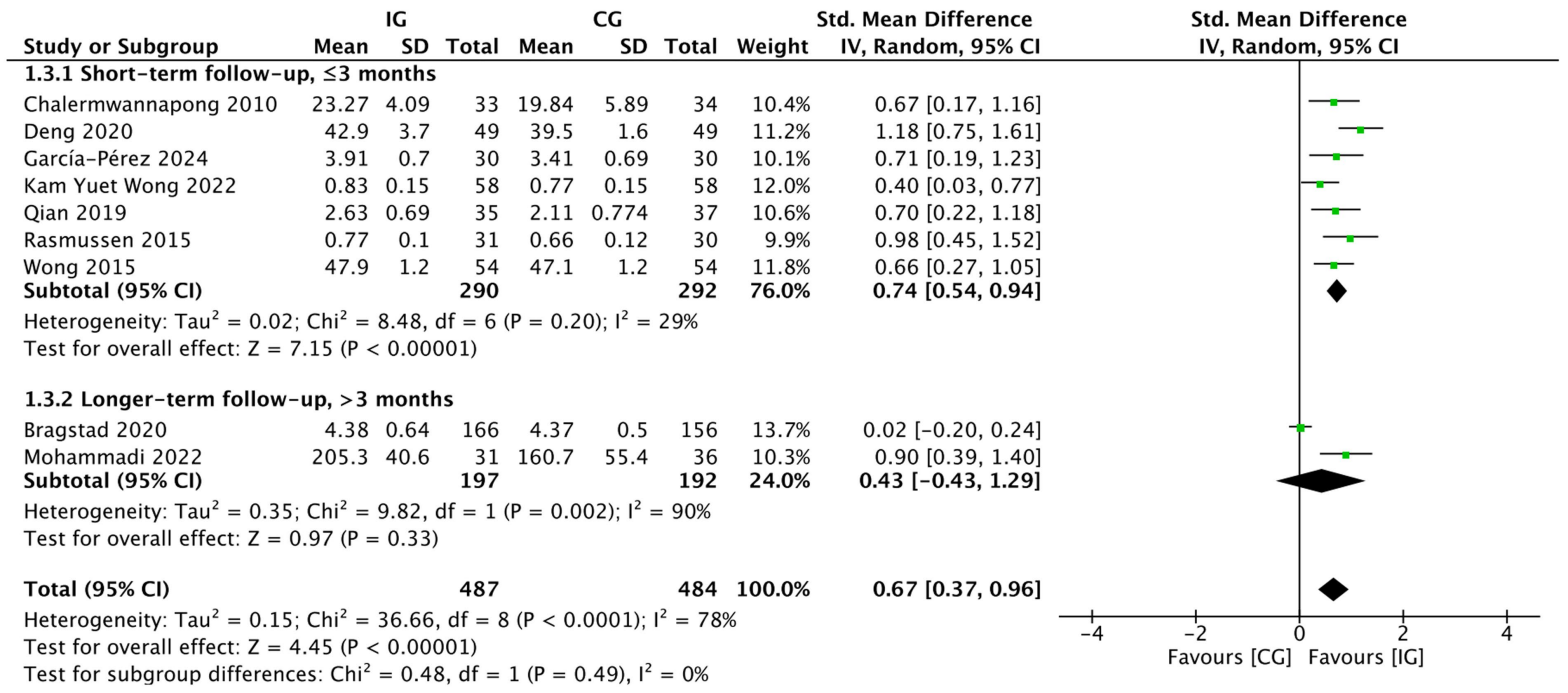
Forest plot of quality of life.

Subgroup analysis showed that the effect was clearer in the short-term follow-up subgroup. Seven trials involving 582 participants reported QoL within 3 months, and transitional care interventions significantly improved short-term QoL (SMD = 0.74, 95% CI 0.54–0.94, *p* < 0.00001), with low-to-moderate heterogeneity (*I*^2^ = 29%). In contrast, two trials involving 389 participants reported longer-term QoL outcomes beyond 3 months, and the pooled estimate was not statistically significant (SMD = 0.43, 95% CI − 0.43 to 1.29, *p* = 0.33), with substantial heterogeneity (*I*^2^ = 90%). The test for subgroup differences was not significant (Figure 6).

These results suggest that transitional care may provide more consistent short-term benefits for QoL, whereas longer-term effects remain uncertain because of the limited number of studies and high heterogeneity.

#### 3.4.4 Mortality

**Figure 7:**
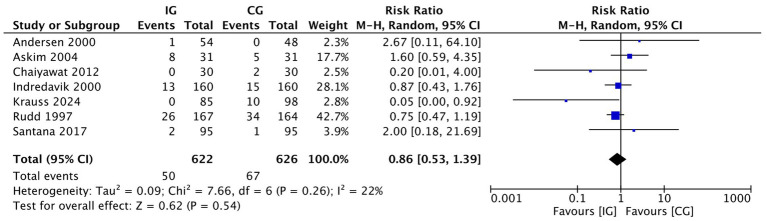
Forest plot of mortality.

Seven randomized controlled trials involving 1,248 participants reported mortality during follow-up periods longer than 3 months. The pooled analysis showed no statistically significant difference in mortality between the transitional care and usual care groups (RR = 0.86, 95% CI 0.53–1.39, *p* = 0.54). Heterogeneity was low (*I*^2^ = 22%). The confidence interval crossed the line of no effect and included both potential benefit and potential harm, indicating that current evidence is insufficient to determine whether transitional care reduces mortality after stroke (Figure 7).

#### 3.4.5 Disability measured by the modified Rankin scale

**Figure 8:**
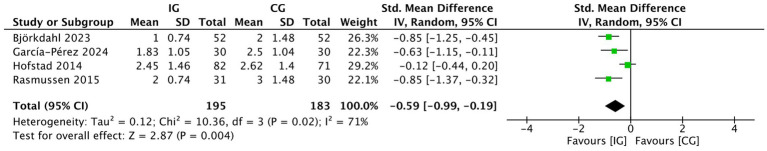
Forest plot of disability measured by the modified Rankin Scale.

Four randomized controlled trials involving 378 participants reported short-term disability measured by the mRS. Transitional care interventions were associated with lower disability scores within 3 months compared with usual care (SMD = −0.59, 95% CI − 0.99 to −0.19, *p* = 0.004). However, heterogeneity was substantial (*I*^2^ = 71%). Because the mRS is an ordinal disability scale and was analyzed as a continuous outcome, this finding should be interpreted cautiously. The result suggests a possible short-term reduction in disability, but the certainty of this evidence is limited by inconsistency and measurement considerations (Figure 8). A summary of the pooled effects for all prespecified meta-analyzed outcomes is presented in Table 2.

**Table 2:** Summary of meta-analysis results.

Outcome	Studies	Participants	Effect	*p*	*I* ^2^	Interpretation
Rehospitalization, ≤3 months	4	507	RR = 0.49, 95% CI 0.29–0.82	0.007	0%	Significant reduction
Rehospitalization, >3 months	3	573	RR = 0.65, 95% CI 0.34–1.21	0.17	70%	Not significant
ADL, overall	16	1,652	SMD = 0.43, 95% CI 0.20–0.67	0.0003	81%	Improved, high heterogeneity
ADL sensitivity	13	1,367	SMD = 0.22, 95% CI 0.07–0.37	0.003	45%	Direction robust
QoL, ≤3 months	7	582	SMD = 0.74, 95% CI 0.54–0.94	<0.00001	29%	Significant improvement
QoL, >3 months	2	389	SMD = 0.43, 95% CI -0.43–1.29	0.33	90%	Not significant
Mortality, >3 months	7	1,248	RR = 0.86, 95% CI 0.53–1.39	0.54	22%	Not significant
mRS, ≤3 months	4	378	SMD = −0.59, 95% CI -0.99 to −0.19	0.004	71%	Lower disability, cautious interpretation
Egger test for ADL sensitivity	13	—	z = 2.518	0.012	—	Possible small-study effects

### 3.5 Publication bias and small-study effects

Small-study effects were assessed for ADL after sensitivity analysis because this outcome included a sufficient number of studies. Visual inspection of the funnel plot suggested possible asymmetry. Egger’s test further indicated potential small-study effects (*z* = 2.518, *p* = 0.012). Therefore, although the beneficial effect of transitional care on ADL remained statistically significant after sensitivity analysis, possible small-study effects should be considered when interpreting the magnitude of the effect (Supplementary Figure S2).

Publication bias assessment was not performed for outcomes with fewer than 10 studies, including rehospitalization, QoL, mortality, and mRS outcomes, because funnel plot asymmetry tests are unreliable when the number of studies is small.

### 3.6 Certainty of evidence

The certainty of evidence for the main outcomes is summarized in Table 3. The certainty of evidence was rated as moderate for short-term rehospitalization and short-term QoL, and low for ADL, mortality, and short-term disability measured by the mRS.

**Table 3:** Summary of findings and certainty of evidence.

Outcome	No. of studies/participants	Effect estimate	Anticipated absolute effect	Certainty of evidence	Interpretation
Rehospitalization within 3 months	4 RCTs / 507 participants	RR = 0.49, 95% CI 0.29 to 0.82	Control risk: 161 per 1,000; intervention risk: 79 per 1,000; 82 fewer per 1,000, from 114 fewer to 29 fewer	⨁⨁⨁◯ Moderate	Transitional care probably reduces short-term rehospitalization.
Activities of daily living	16 RCTs / 1,652 participants	SMD = 0.43, 95% CI 0.20 to 0.67	Sensitivity analysis: SMD = 0.22, 95% CI 0.07 to 0.37	⨁⨁◯◯ Low	Transitional care may improve ADL, but certainty is reduced by heterogeneity and possible small-study effects.
Quality of life within 3 months	7 RCTs / 582 participants	SMD = 0.74, 95% CI 0.54 to 0.94	Not applicable for SMD outcome	⨁⨁⨁◯ Moderate	Transitional care probably improves short-term quality of life.
Mortality at >3 months follow-up	7 RCTs / 1,248 participants	RR = 0.86, 95% CI 0.53 to 1.39	Control risk: 107 per 1,000; intervention risk: 92 per 1,000; 15 fewer per 1,000, from 50 fewer to 42 more	⨁⨁◯◯ Low	The effect of transitional care on mortality is uncertain.
Disability measured by mRS within 3 months	4 RCTs / 378 participants	SMD = −0.59, 95% CI -0.99 to −0.19	Not applicable for SMD outcome	⨁⨁◯◯ Low	Transitional care may reduce disability, but interpretation should be cautious.

GRADE certainty levels: high, moderate, low, and very low. a. Rehospitalization: downgraded one level for risk of bias because blinding of participants and personnel was generally not feasible in transitional care interventions, although rehospitalization is an objective outcome.b. ADL: downgraded for risk of bias because ADL is a subjective or assessor-dependent outcome and blinding was frequently not feasible. Downgraded for inconsistency because heterogeneity was substantial in the primary analysis (*I*^2^ = 81%), although sensitivity analysis reduced heterogeneity to 45%. Egger’s test suggested possible small-study effects (*p* = 0.012), which was considered when interpreting the certainty of evidence.c. Quality of life: downgraded one level for risk of bias because quality of life is a patient-reported outcome and participant blinding was generally not feasible. d. Mortality: downgraded two levels for imprecision because the confidence interval crossed the line of no effect and included both potential benefit and harm. e. mRS: downgraded for risk of bias and inconsistency because mRS is assessor-dependent and heterogeneity was substantial (*I*^2^ = 71%). The ordinal mRS scale was analyzed as a continuous outcome; therefore, results should be interpreted cautiously.

For short-term rehospitalization, the certainty of evidence was downgraded to moderate for risk of bias because blinding of participants and personnel was generally not feasible in transitional care interventions, although rehospitalization was an objective outcome. For ADL, the certainty of evidence was rated as low because of risk of bias, substantial heterogeneity in the primary analysis, and possible small-study effects. For short-term QoL, the certainty of evidence was moderate, mainly downgraded for risk of bias because QoL was patient-reported and participant blinding was generally not feasible. For mortality, the certainty of evidence was low because of imprecision, as the confidence interval crossed the line of no effect and included both potential benefit and potential harm. For disability measured by the mRS, the certainty of evidence was low because of risk of bias, substantial heterogeneity, and the limitations of analyzing an ordinal scale as a continuous outcome. Detailed reasons for downgrading are provided in the footnotes of the Summary of Findings table to ensure that the strength of each conclusion is aligned with the certainty of the underlying evidence (Table 3).

## 4 Discussion

This updated systematic review and meta-analysis synthesized evidence from 24 randomized controlled trials involving 3,520 stroke survivors and found that transitional care interventions were associated with a lower risk of rehospitalization, particularly within the first 3 months after discharge. The evidence also suggested improvements in ADL and QoL, although the certainty and stability of these findings differed across outcomes. The short-term QoL result was relatively consistent, whereas the ADL effect was smaller after sensitivity analysis and remained vulnerable to heterogeneity and possible small-study effects. No significant reduction in mortality was observed, and the apparent short-term improvement in mRS scores should be regarded as exploratory because the mRS is an ordinal scale and the analysis was based on a limited number of studies. Overall, the findings indicate that transitional care is most likely to benefit early post-discharge outcomes that are directly influenced by care continuity, monitoring, rehabilitation support, and access to timely professional guidance.

The reduction in early rehospitalization is clinically plausible. The first weeks and months after stroke discharge are characterized by rapid changes in functional status, medication adjustment, rehabilitation needs, caregiver burden, and uncertainty about when to seek medical help. Transitional care may reduce avoidable rehospitalization by extending clinical oversight beyond discharge, reinforcing medication and vascular risk-factor management, identifying complications earlier, and improving linkage with rehabilitation and community services. These mechanisms are consistent with the broader transitional care model, in which structured discharge planning and post-discharge follow-up are intended to maintain continuity and prevent care fragmentation during high-risk transitions (6, 14). The fact that the effect was clearer within 3 months than beyond 3 months suggests that transitional care may primarily act on early, transition-sensitive risks rather than on longer-term events driven by recurrent vascular disease, comorbidity burden, socioeconomic factors, or healthcare-system access. Accordingly, an absence of a statistically significant longer-term effect should not be interpreted as proof of no benefit; rather, the available evidence remains uncertain and may indicate that time-limited transitional programs need to be integrated with sustained secondary prevention and community rehabilitation to influence later outcomes.

The findings for ADL require more cautious interpretation. Although the primary analysis suggested functional benefit, heterogeneity was substantial and the effect estimate decreased after sensitivity analysis. This pattern implies that the initial pooled estimate may have been influenced by study-level factors such as unusually large effects, small sample sizes, differences in intervention intensity, or variation in measurement instruments. ADL was measured using different scales, including the Barthel Index, Modified Barthel Index, Functional Independence Measure, and related tools, which differ in scoring range, responsiveness, and assessor dependence. The interventions also differed substantially: some included active home-based rehabilitation, task-oriented practice, or caregiver-supported exercises, whereas others emphasized education, telephone follow-up, or care coordination. These differences are clinically meaningful because functional recovery after stroke is strongly influenced by the dose, timing, and task specificity of rehabilitation, as well as by baseline disability and caregiver support (2). Therefore, the ADL result should be interpreted as evidence of a possible functional benefit rather than as confirmation of a large or uniform effect across all transitional care models.

QoL appeared to improve more consistently in the short term. This may reflect the multidimensional nature of QoL after stroke, which is shaped not only by physical function but also by perceived safety, emotional adjustment, confidence in self-management, caregiver support, communication with healthcare professionals, and the availability of services after discharge. Transitional care may improve QoL by reducing uncertainty, providing a reliable point of contact, clarifying rehabilitation goals, and helping survivors and caregivers translate discharge instructions into daily practice. The more consistent short-term QoL finding also suggests that survivors may experience benefit from improved support and continuity even when measurable functional gains are modest. However, the longer-term QoL evidence remains limited. Sustained QoL improvement is likely to depend on continued rehabilitation, psychosocial support, social participation, and secondary prevention beyond the initial transition period.

The absence of a statistically significant mortality effect should be interpreted in the context of imprecision rather than as definitive evidence of no effect. Mortality after stroke is determined by age, stroke severity, comorbidities, recurrent vascular events, acute treatment, and the quality of long-term secondary prevention. Most included transitional care interventions were not primarily designed or powered to reduce mortality, and the confidence interval included both potential benefit and potential harm. Similarly, the mRS finding suggests a possible short-term disability benefit, but the evidence is limited by heterogeneity, small numbers of studies, and the analytical treatment of an ordinal outcome as a continuous measure. Future trials should report mRS using appropriate ordinal or prespecified dichotomous approaches and should define clinically meaningful thresholds in advance.

Our findings are broadly consistent with previous evidence suggesting that organized post-discharge stroke services, early supported discharge, and transitional care may improve selected functional or service-use outcomes. The Cochrane review of early supported discharge found that coordinated multidisciplinary services can reduce dependency and length of hospital stay, especially among stroke survivors with mild-to-moderate disability (15). More recent reviews of transitional care services have also reported potential benefits for functional outcomes, QoL, and readmission-related outcomes (8, 9). However, the present review differs in several important respects. It updates the evidence base to May 13, 2026; separates clinical, functional, and QoL outcomes to avoid treating ADL as a patient-reported outcome; explores follow-up duration as a clinically relevant source of heterogeneity; applies sensitivity analysis and small-study-effect assessment where appropriate; and grades the certainty of evidence using GRADE. These features allow a more conservative interpretation of the evidence and help distinguish outcomes with relatively stable support from those that remain uncertain (Supplementary Table S3).

The certainty of evidence is central to the interpretation of this review. Evidence for short-term rehospitalization and short-term QoL was rated as moderate, supporting a probable benefit of transitional care for these outcomes. In contrast, evidence for ADL, mortality, and mRS was rated as low because of risk of bias, inconsistency, imprecision, or possible small-study effects. These ratings mean that the direction of effect for some outcomes may be promising, but the magnitude of benefit remains uncertain. In particular, the ADL finding should not be overinterpreted because Egger’s test suggested possible small-study effects, and the sensitivity analysis produced a smaller estimate. The findings are also not equally generalizable across all health systems. Included studies were conducted in different countries and service contexts, where usual care, access to rehabilitation, community resources, insurance coverage, and caregiver roles may differ substantially. Therefore, implementation should be adapted to local care pathways rather than directly transferred as a uniform model.

This review has several strengths. It followed PRISMA 2020 guidance, used a registered protocol, updated the search to May 2026, and included only randomized controlled trials. Study selection, data extraction, and risk-of-bias assessment were performed independently by reviewers. The analysis distinguished clinical, functional, and QoL outcomes; explored follow-up duration; conducted sensitivity analysis for the most heterogeneous major outcome; assessed small-study effects when the number of studies was sufficient; and used GRADE to align conclusions with the certainty of evidence. These methodological steps directly address several limitations of earlier syntheses and reduce the risk of overstatement.

Clinically, the results support transitional care as a structured early discharge-to-home strategy rather than as a stand-alone solution for all long-term stroke outcomes. Programs aiming to reduce early rehospitalization should prioritize individualized discharge planning, early follow-up, medication and risk-factor review, rapid response to emerging problems, and coordination with rehabilitation and community services. Programs aiming to improve ADL should include active rehabilitation components, task-oriented practice, and caregiver-supported exercises rather than relying solely on education or follow-up calls. Future research should use multicenter, adequately powered randomized designs with longer follow-up, standardized outcome definitions, detailed intervention reporting, and prespecified economic evaluation. The Template for Intervention Description and Replication (TIDieR) framework may help improve the reporting of intervention components and support replication across settings (16). Future evidence syntheses may also benefit from component-based meta-analysis, network meta-analysis, or individual participant data meta-analysis to identify which intervention components, delivery models, and patient subgroups are most likely to benefit.

## 5 Limitations

The main limitations of this review relate to intervention complexity, study quality, outcome measurement, and evidence precision. Transitional care interventions varied substantially across trials in provider type, delivery mode, duration, intensity, rehabilitation content, caregiver involvement, and community linkage. This clinical heterogeneity limited the ability to determine the independent contribution of specific intervention components. Although follow-up-based subgroup analysis and ADL sensitivity analysis were conducted, the available data were insufficient for reliable meta-regression or component-level inference.

Methodological limitations of the included trials may also have influenced the findings. Blinding of participants and personnel was generally not feasible, and incomplete outcome data or unclear reporting was present in several studies. These issues are particularly relevant for subjective or assessor-dependent outcomes, including ADL, QoL, and disability. In addition, outcome instruments were not standardized across studies, and some scales differed in scoring direction, responsiveness, and clinical interpretation.

Several outcomes were supported by a limited number of trials or participants. Mortality and mRS findings were imprecise, and longer-term QoL evidence was sparse. ADL results may have been affected by possible small-study effects, as suggested by funnel plot asymmetry and Egger’s test. Therefore, although the direction of effect was generally favorable, the magnitude of benefit for functional outcomes should be interpreted cautiously.

This review included only English-language reports, which may have introduced language bias. Despite supplementary searches, unpublished negative trials may also have been missed. Finally, most included studies did not provide sufficient information on intervention cost, implementation fidelity, or health-system context. As a result, this review supports the probable value of transitional care for selected early post-discharge outcomes but cannot determine the optimal intervention model, dose, cost-effectiveness, or scalability across different healthcare settings.

## 6 Conclusion

Transitional care interventions probably reduce short-term rehospitalization and improve short-term QoL in stroke survivors. They may also improve ADL and short-term disability, but these findings should be interpreted cautiously because of heterogeneity, risk of bias, and possible small-study effects. Current evidence does not establish a clear mortality benefit. Future studies should define intervention components more precisely, evaluate long-term and clinically meaningful outcomes, and determine which transitional care models are most effective, scalable, and cost-effective across healthcare settings.

## Data Availability

The original contributions presented in the study are included in the article/Supplementary material, further inquiries can be directed to the corresponding author/s.
