# Supplementary figures and images for: Effects of transitional care interventions on rehospitalization, functional outcomes, and quality of life in stroke survivors: an updated systematic review and meta-analysis of randomized controlled trials
Source: Front Neurol. 2026 Jun 23;17:1769301. doi: 10.3389/fneur.2026.1769301 (PMC13337450; doi:10.3389/fneur.2026.1769301)

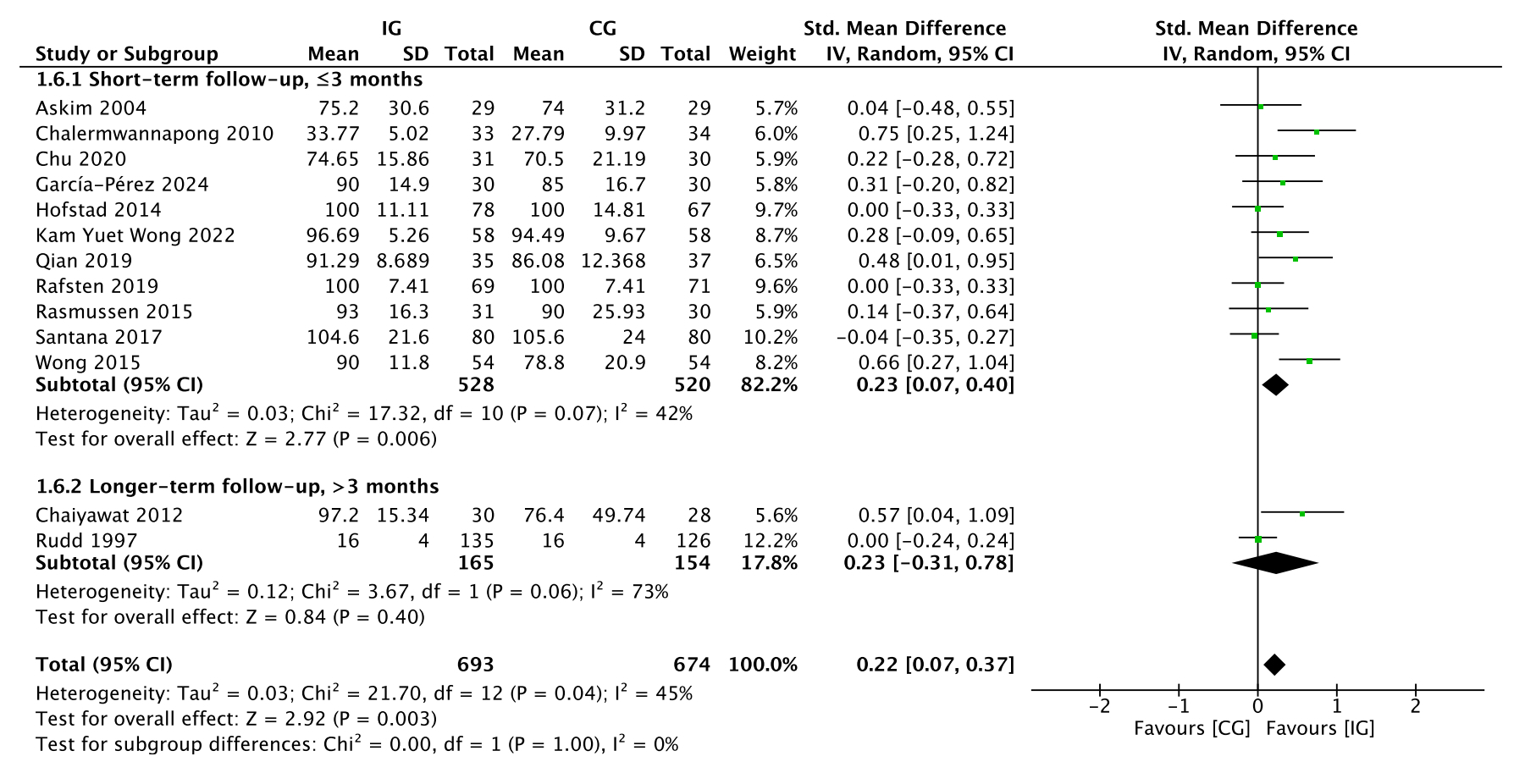

Supplement: Supplementary file 1 [file Image_1.JPEG]

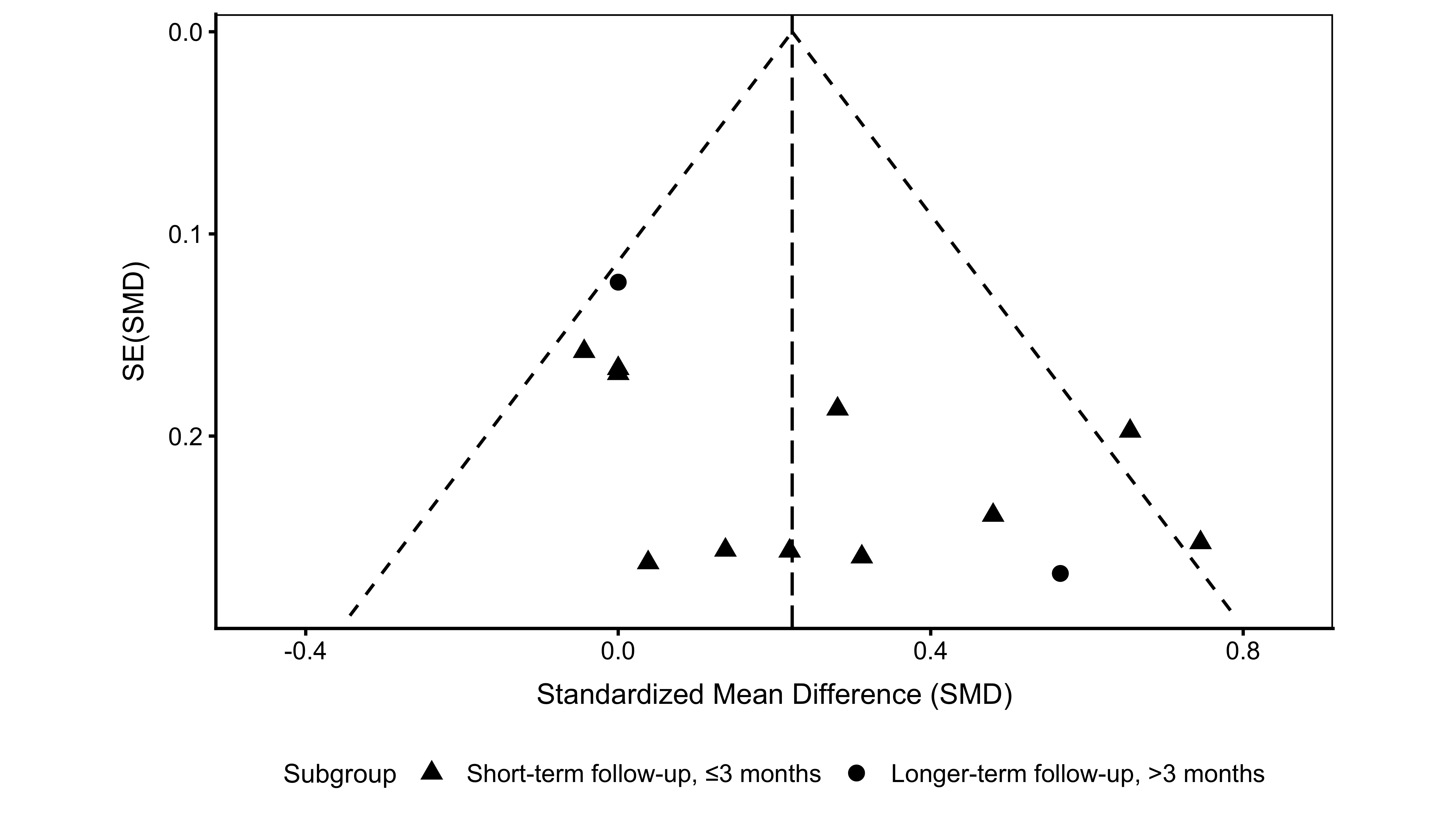

Supplement: Supplementary file 2 [file Image_2.JPEG]
