# Supplementary material for: Effects of transitional care interventions on rehospitalization, functional outcomes, and quality of life in stroke survivors: an updated systematic review and meta-analysis of randomized controlled trials
Source: Front Neurol. 2026 Jun 23;17:1769301. doi: 10.3389/fneur.2026.1769301 (PMC13337450; doi:10.3389/fneur.2026.1769301)
Supplement: Supplementary file 3 [file Table_1.DOCX]

**Table S1. Search strategies**

| Keywords | Search strategy | Results |
| --- | --- | --- |
| **Pubmed** |  |  |
| #1 Stroke | "stroke"[MeSH Terms] OR "stroke"[Title/Abstract] OR "ischemic stroke"[Title/Abstract] OR "hemorrhagic stroke"[Title/Abstract] OR "cerebral infarction"[Title/Abstract] OR "brain infarction"[Title/Abstract] OR "cerebral hemorrhage"[Title/Abstract] | 435,665 |
| #2 Transitional care | "transitional care"[MeSH Terms] OR "transitional care"[Title/Abstract] OR "care transition"[Title/Abstract] OR "hospital to home"[Title/Abstract] OR "post discharge care"[Title/Abstract] OR "supported discharge"[Title/Abstract] | 7,327 |
|  | #1 and #2 | 521 |

**Table S1. Search strategies (continues)**

| Keywords | Search strategy | Results |
| --- | --- | --- |
| **Web of science** |  |  |
| #1 Stroke | TS=("stroke" OR "ischemic stroke" OR "hemorrhagic stroke" OR "cerebral infarction" OR "brain infarction" OR "cerebral hemorrhage") | 512,828 |
| #2 Transitional care | TS=("transitional care" OR "care transition" OR "hospital to home" OR "post discharge care" OR "supported discharge") | 6,915 |
|  | #1 and #2 | 739 |

**Table S1. Search strategies (continues)**

| Keywords | Search strategy | Results |
| --- | --- | --- |
| **Embase** |  |  |
| #1 Cerebrovascular accident | 'cerebrovascular accident'/exp OR 'stroke':ti,ab,kw OR 'ischemic stroke':ti,ab,kw OR 'hemorrhagic stroke':ti,ab,kw OR 'cerebral infarction':ti,ab,kw OR 'brain infarction':ti,ab,kw OR 'cerebral hemorrhage':ti,ab,kw | 800,342 |
| #2 Transitional care | 'transitional care'/exp OR 'transitional care':ti,ab,kw OR 'care transition':ti,ab,kw OR 'hospital to home':ti,ab,kw OR 'post discharge care':ti,ab,kw OR 'supported discharge':ti,ab,kw | 15,264 |
|  | #1 and #2 | 1,333 |

**Table S1. Search strategies (continues)**

| Keywords | Search strategy | Results |
| --- | --- | --- |
| **Cochrane Library** |  |  |
| #1 Stroke | MeSH descriptor: [Stroke] explode all trees OR ("stroke"):ti,ab,kw OR ("ischemic stroke"):ti,ab,kw OR ("hemorrhagic stroke"):ti,ab,kw OR ("cerebral infarction"):ti,ab,kw OR ("brain infarction"):ti,ab,kw OR ("cerebral hemorrhage"):ti,ab,kw | 86,100 |
| #2 Transitional Care | MeSH descriptor: [Transitional Care] explode all trees OR ("transitional care"):ti,ab,kw OR ("care transition"):ti,ab,kw OR ("hospital to home"):ti,ab,kw OR ("post discharge care"):ti,ab,kw OR ("supported discharge"):ti,ab,kw | 1,541 |
|  | #1 and #2 | 275 |

**Table S1. Search strategies (continues)**

| Keywords | Search strategy | Results |
| --- | --- | --- |
| **CINAHL** |  |  |
| #1 Stroke | XB ("stroke" OR "ischemic stroke" OR "hemorrhagic stroke" OR "cerebral infarction" OR "brain infarction" OR "cerebral hemorrhage") | 124,830 |
| #2 Transitional care | XB ("Transitional care" OR "Cares, Transitional" OR "Care, Transitional" OR "Transitional Cares" OR "Transition Care" OR "Transition Cares" OR "Home Transition" OR "Home Transitions" OR "Transition, Home" OR "Transitions, Home") | 4,368 |
|  | #1 and #2 | 136 |
