# Supplementary material for: Effects of transitional care interventions on rehospitalization, functional outcomes, and quality of life in stroke survivors: an updated systematic review and meta-analysis of randomized controlled trials
Source: Front Neurol. 2026 Jun 23;17:1769301. doi: 10.3389/fneur.2026.1769301 (PMC13337450; doi:10.3389/fneur.2026.1769301)
Supplement: Supplementary file 4 [file Table_2.DOCX]

**Table S2 Intervention-component matrix**

| Study | Intervention category | Start time | Intervention duration | Contact frequency/dose | Home visit | Telephone/digital follow-up | Rehabilitation training | Caregiver involvement | Case management/referral | Theory or framework |
| --- | --- | --- | --- | --- | --- | --- | --- | --- | --- | --- |
| (Krauss et al., 2024)  USA | Home-based transition intervention | Pre-discharge | 5 months | Mean 3–4 visits; 2 booster visits | Yes | No | Partial | NR | No | COMPASS; I-HOPE model |
| (García-Pérez et al., 2024)  ESP | Occupational therapy transition program | Pre-discharge | 3 months | 1 hospital session; 1 home visit; weekly calls | Yes | Telephone | Yes | Yes | Partial | NR |
| (Li et al., 2024)  CHN | Extended post-discharge care | During hospitalization | 3 months | Alternating home visits and calls | Yes | Telephone | Yes | Yes | Yes | Continuous care model |
| (Björkdahl et al., 2023)  SWE | Very early supported discharge | Pre-discharge | 4 weeks | 2–4 visits/week | Yes | NR | Yes | NR | Partial | Early supported discharge model |
| (Kam Yuet Wong et al., 2022)  CHN | Home-based transitional care | Pre-discharge | 12 weeks | 1 pre-discharge session; 6 home visits; 6 calls | Yes | Telephone | Yes | Yes | Yes | Transitional care framework; Omaha System |
| (Lin et al., 2022)  CHN | Nurse-led health coaching | Pre-discharge | 12 weeks | Weekly calls; biweekly coaching | No | Telephone | Yes | Yes | Partial | Self-efficacy theory |
| (Mohammadi et al., 2022)  IRN | Partnership care model | Post-discharge | 6 months | 3 weekly sessions; monthly visits | No | No | Partial | Yes | Yes | Partnership Care Model |
| (Deng et al., 2020)  CHN | Integrated transitional care | Post-discharge | 8 weeks | 3 home visits/week; ≥2 calls/week | Yes | Telephone | Yes | NR | Yes | Integrated transitional care model |
| (Chu et al., 2020)  CHN | Family-delivered rehabilitation | Pre-discharge | 8 weeks | 3 training sessions; follow-up calls | No | Telephone + digital | Yes | Yes | No | Family-delivered rehabilitation model |
| (Bragstad et al., 2020)  NOR | Psychosocial support intervention | 4–8 weeks post-stroke | 6 months | 8 sessions | Yes | NR | No | NR | No | Guided self-determination |
| (Rafsten et al., 2019)  SWE | Very early supported discharge | Pre-discharge | 4 weeks | 2–4 visits/week | Yes | NR | Yes | Yes | Partial | Person-centred goal-setting |
| (Qian et al., 2019)  CHN | Transitional nursing care | Pre-discharge | 5 weeks | 1 education session; 1 call; 1 home visit | Yes | Telephone | Yes | NR | Yes | Omaha-based nursing care |
| (Chen et al., 2018)  CHN | Self-management empowerment | During hospitalization | 6 weeks | 5 bedside sessions; 1 group session; 4 calls | No | Telephone | Yes | Yes | Partial | Health empowerment model |
| (Santana et al., 2017)  POR | Early home-supported discharge | Pre-discharge | 1 month | Approximately 8 home sessions | Yes | NR | Yes | Yes | Yes | Early home-supported discharge model |
| (Wong and Yeung, 2015)  CHN | Nurse-led transitional care | Pre-discharge | 4 weeks | Weekly home visits; weekly calls | Yes | Telephone | Yes | Yes | Yes | Omaha System; 4C model |
| (Rasmussen et al., 2016)  DEN | Home-based rehabilitation | During hospitalization | 4 weeks | 1–3 visits/week before discharge; 1–5 days/week after discharge | Yes | NR | Yes | Yes | Yes | Home-based rehabilitation model |
| (Hofstad et al., 2014)  NOR | Early supported discharge | During hospitalization | 5 weeks | Up to 4 h/day; 5 days/week | No | NR | Yes | Yes | Yes | Early supported discharge model |
| (Chaiyawat and Kulkantrakorn, 2012)  THA | Home rehabilitation program | Post-discharge | 6 months | Monthly home visits | Yes | Telephone | Yes | Yes | NR | Motor learning framework |
| (Chalermwannapong et al., 2010)  THA | Transitional care program | During hospitalization | 4 weeks | Daily hospital visits; 2 home visits; 2 calls | Yes | Telephone | Yes | Yes | Yes | Naylor Transitional Care Model |
| (Allen et al., 2009)  USA | Postdischarge care management | Post-discharge | 6 months | 1 home assessment; weekly calls initially; additional visits as needed | Yes | Telephone | Partial | NR | Yes | Chronic Care Model |
| (Askim et al., 2004)  NOR | Extended stroke unit service | During hospitalization | 4 weeks | At least 1 home visit; telephone follow-up; outpatient consultation | Yes | Telephone | Yes | Yes | Yes | Extended stroke unit service |
| (Indredavik et al., 2000)  NOR | Extended stroke unit service | During hospitalization | 1 month | Individualized home visit and follow-up | Yes | Telephone | Yes | Yes | Yes | Early supported discharge model |
| (Andersen et al., 2000)  DEN | Physician-led follow-up | Post-discharge | 12 weeks | 3 home visits | Yes | Telephone | Partial | Yes | Yes | Physician-led follow-up model |
| (Rudd et al., 1997)  GBR | Early discharge scheme | Post-randomization | 3 months | Domiciliary therapy as needed | Yes | NR | Yes | Yes | Yes | Community stroke rehabilitation model |
