# Supplementary material for: Effects of transitional care interventions on rehospitalization, functional outcomes, and quality of life in stroke survivors: an updated systematic review and meta-analysis of randomized controlled trials
Source: Front Neurol. 2026 Jun 23;17:1769301. doi: 10.3389/fneur.2026.1769301 (PMC13337450; doi:10.3389/fneur.2026.1769301)
Supplement: Supplementary file 5 [file Table_3.DOCX]

Table S3 Methodological comparison between the present review and previous systematic reviews.

| Review | Main focus | Key limitations relevant to the present review | Added value of the present review |
| --- | --- | --- | --- |
| (Wang et al., 2017) | Transitional care and selected clinical or functional outcomes after stroke | Earlier evidence base; limited incorporation of more recent randomized trials; limited exploration of newer delivery models | Updated evidence base; refined outcome domains; follow-up-based subgroup analysis; GRADE assessment |
| (Kim et al., 2025) | Transitional care services for patients with stroke | Recent synthesis, but outcome classification and certainty interpretation may not fully address ADL as a functional or assessor-dependent outcome | Clear separation of clinical, functional, and QoL outcomes; cautious interpretation according to GRADE certainty |
| (Tan et al., 2026) | Multidisciplinary transitional care interventions and outcomes such as functional status, QoL, and rehospitalization | Focused on multidisciplinary models; residual uncertainty regarding follow-up duration and heterogeneity across outcomes | Inclusion of broader transitional care models; follow-up-based subgroup analyses; sensitivity analysis for ADL; small-study-effect assessment where appropriate |
| Present review | Transitional care interventions for rehospitalization, functional outcomes, QoL, mortality, and disability in stroke survivors | Remaining limitations include intervention heterogeneity and limited data for component-level inference | Updated RCT synthesis; explicit protocol amendments; structured outcome classification; sensitivity and small-study-effect assessment; GRADE-based interpretation |
